# Supplementary material for: Self-sustained non-equilibrium co-existence of fluid and solid states in a strongly coupled complex plasma system
Source: Sci Rep. 2022 Aug 16;12:13882. doi: 10.1038/s41598-022-17939-w (PMC9381532; doi:10.1038/s41598-022-17939-w)
Supplement: Supplementary file 1 — Supplementary Information. [file 41598_2022_17939_MOESM1_ESM.pdf]

Supplementary materials

General framework of the simulations

The experimentally observed complex plasmas system is non-equilibrium, non-uniform, finite-sized and comprises regions with different geometry. Three-dimensional multilayer central subsystem is in contact with the quasi-two-dimensional single-layered peripheral subsystem. The latter one is "quasi"-2D, because the motion of its particles in the vertical direction is present as well. The term "phase" is applied to systems in the range of states from thermodynamically equilibrium to non-equilibrium metastable ones. As soon as the considered experimental system of microparticles is stable and is in partial equilibrium, we apply the term "phase coexistence" to describe its state meaning the coexistence of ordered and disordered states in the structure.

In order to describe the process of non-equilibrium phase coexistence observed in the experimental system, it is necessary to include the correct mechanism of continuous energy input that heats the central part of the system and leads to its non-equilibrium melting. As soon as melting starts in the structure only after reorganization of particles into the second layer in the central region, the effect of laser radiation on the dynamics of particles might be regarded as small. Then the main candidate for the energy source is the effectively nonreciprocal and nonconservative interaction of particles with the plasma wakes. There are several extensively used approaches to the simulation of dust particle dynamics in presence of the wake effect.

The simplified approach most commonly used in simulations employs the point charge approximation of the plasma wakes[1–5]. Within this approach, each plasma wake can be considered as a point-like positive charge q located strictly below a dust particle at a fixed distance δ_p. Such approach, although very successful, intuitive and resource-efficient, does not allow direct calculation of the parameters q and δ_p self-consistently using the experimental plasma parameters. Therefore, it pretends only for the qualitative analysis of experimental data.

More accurate methods involve the self-consistent calculation of ion kinetics in the electric field near charged dust grains[6–8]. Such methods are resource-intensive and best applied to the systems of one or two grains. The recently developed numerical model "Dynamic Response of Ions and Dust" (DRIAD)[9] employs the concept of superions with the same charge-to-mass ratio as a single ion and allows to treat the wake formation and grain charging in a self-consistent manner. The model demonstrates good predictive ability in determination of grain charges in the linear vertical chain comprising seven dust particles.

In the present work, the experimental system consists of more than 2000 dust particles. The structure is large enough to exhibit macroscopic behavior[10,11]. Simulations of phase coexistence in the system of this size impose certain limitations on the numerical technique. On the one hand, it should allow efficient calculation of the many-body system, on the other hand, it should provide more accurate description of the wake structure in the flowing plasmas than the point-wake model. The calculation scheme applied in the present work is described further.

Equations of dust particles motion are solved numerically with the preset interaction potential, as in the classical molecular dynamics (MD) method. In contrast to the theoretical works where the interaction of dust particles is given by the Yukawa-like potential and the point-like ion focus term[4,5], we employ the particle-in-cell method for the explicit calculation of ions kinetics and the electrostatic potential around a dust particle[6,7,12]. Such an approach to the calculation of the electrostatic potential, as stated above, is self-consistent and allows to exclude additional parameters of the ion focus from the mathematical model. The interaction potential in this case can be calculated via the plasma parameters obtained from the experiment.

For the efficiency of simulations, the distribution of electrostatic potential around dust particles is assumed to be unchanged during the simulation of dust dynamics and is calculated at the initial step of the simulation. Calculation of the potential is done for a solitary dust particle at the parameters of plasma close to the experimental ones. Forces acting on the particles are calculated under the following assumptions:

1. The electric charge of all particles Q is equal and does not change during the molecular dynamics simulation.
2. The electric potential in the system can be represented as the linear superposition of the individual dust particles potentials and the electrostatic trap.
3. Relaxation time of the distribution of ions and of the electrostatic potential under the change of dust particles positions is much smaller than the minimum characteristic time of a dust particle dynamics.
4. Neighboring particles weakly change the trajectories of ions near positions of each other.

Under these assumptions, the interparticle interaction force is calculated via the gradient of the electrostatic potential in the system. The interaction of particles with the perturbed ion flow, i.e., the ion drag force[7,13], is assumed to be independent of the time and positions of particles. It is disregarded in the calculation of particles dynamics.

The presented approach to the multiscale consideration of the system neglects several effects. Such effects are the dependence of grain charge on the grain position in the wakes of other grains, the dependence of the wake potential itself on the presence of other grains and the nonuniform distribution of plasma parameters in the sheath. Thus, this approach pretends only for the qualitative agreement with the experiment. However, it has a reasonable advantage over the simplified point-wake model

due to the direct account for the experimental plasma parameters. The approach is sufficient to study the observed scenario of phase co-existence in the system.

PIC calculation of the wake potential

We now describe the scheme of calculation of the electrostatic potential around a single dust particle. The potential is calculated self-consistently by the particle-in-cell simulation of ion kinetics[14] taking into account an external electric field and ion-neutral collisions through the resonant charge-exchange mechanism[15]. This mechanism dominates in the gas discharge plasma at low pressures[16]. The inclusion of the external electric field and ion-neutral collisions leads to the deviation of the ion distribution function from the Maxwellian distribution. This deviation changes the structure of the tail of the electrostatic potential around a dust particle[17,18]. It is assumed that electrons have the Boltzmann distribution. In addition, the potential is calculated with the point charge model for a dust particle. The system of equations for the simulation of ion kinetics has the following form in CGS-ESU units:

$$\nabla^2\varphi = -4\pi|e|\left[n_i - n_e\exp\left(-\frac{e\varphi}{k_B T_e}\right) + Q\delta(r)\right], \tag{1}$$

$$\frac{\partial f}{\partial t} + v\nabla f + \frac{|e|}{m_i}\left(E_0 - \nabla\varphi\right)\frac{\partial f}{\partial v} = \omega_{\text{in}}\left(\Phi_M(v)n_i - f\right), \tag{2}$$

where φ is the electrostatic potential, $\Phi_M(v)$ is Maxwellian distribution function of neutrals, e is the negative charge of the electron, k_B is the Boltzmann constant, $\delta(r)$ is the Dirac delta function, m_i is the mass of an ion, E_0 is the external electric field, ν_{in} is the ion-neutral collision frequency, T_e and n_e is the temperature and concentration of electrons at infinity, $f \equiv f(v)$ is the ion distribution function, and n_i is the ion concentration. The system of the Poisson-Boltzmann equation (1) and kinetic equation (2) describes the evolution of ion the distribution function in the self-consistent electrostatic potential. This system of equations is solved numerically using the COPTIC code[19] to obtain the stationary potential distribution around a single dust particle.

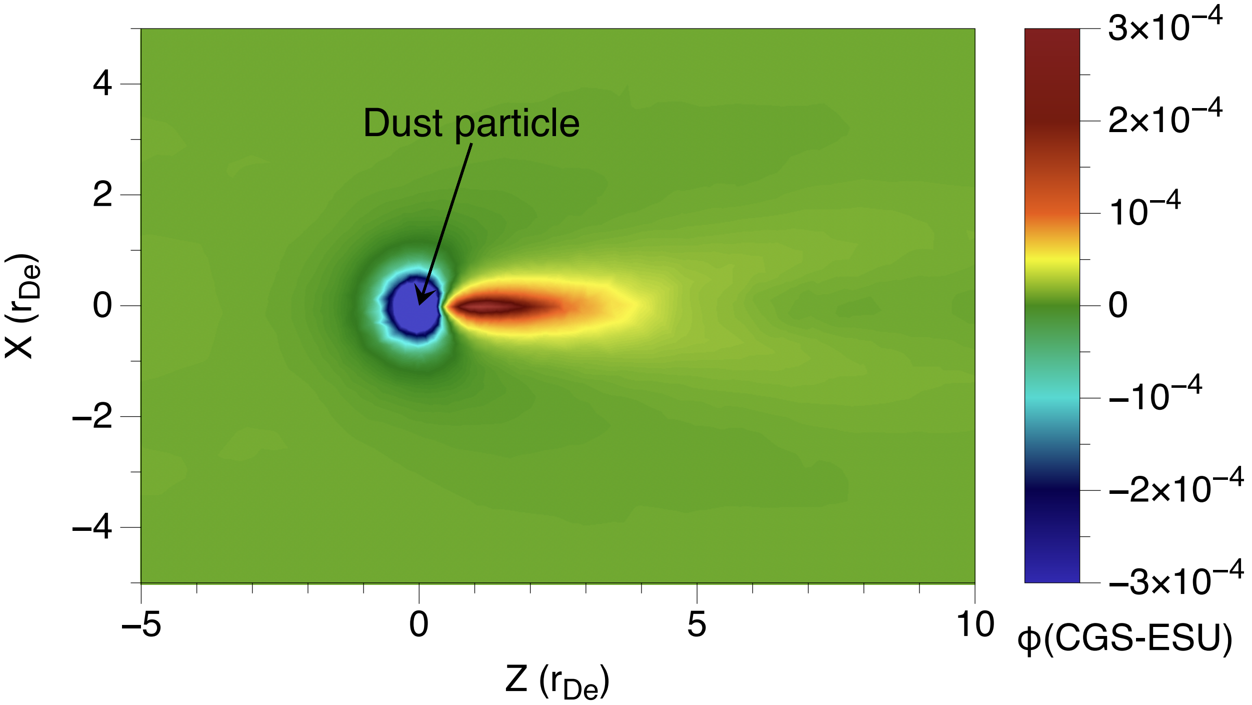

Figure S1. Typical view of anisotropic electrostatic potential map around a single dust particle in the gas discharge plasma. The ion flow is directed from left to right.

The map of one of the calculated electrostatic potentials is shown in Fig. S1. The potential near the dust particle is negative. At the same time, the region of a positive potential appears behind the dust particle in the direction of the ion flow because of ion focusing. The existing region of a positive potential or a so called plasma wake leads to effective nonreciprocity in

interaction between dust particles. Due to such effective nonreciprocity the energy at the dust particles subsystem is generally not conserved. It is explained by the transfer of energy between the plasma and the dust subsystems.

The set of plasma parameters used for calculations of the wake potential under conditions on the experiment is discussed bellow. Electron temperature T_e and ion concentration far from the dust particle n_i^∞ are taken equal to 2.6 eV and 2×10^{15} m^{-3}. These values correspond to our measurements of plasma parameters in the experimental set-up using single and double Langmuir probes as well as emissive probes.

The self-consistent calculation of the charge of all dust particles in the considered many dust particle system is resource-intensive. For this reason, we make several estimations of a dust particle charge. The orbital motion limited (OML) theory[20] provides the value $Q = 21000\ e$ under experimental conditions. However, ion shadowing effects[8] and collisions[21] can lead to decreasing of the estimated charge value by 25-30%. That is why, in the scope of this work, we consider the values of grain charge in the range from $Q = 15000\ e$ to $Q = 21000\ e$. The external electric field E_0 is chosen to balance the gravitational force acting on the charged dust particles.

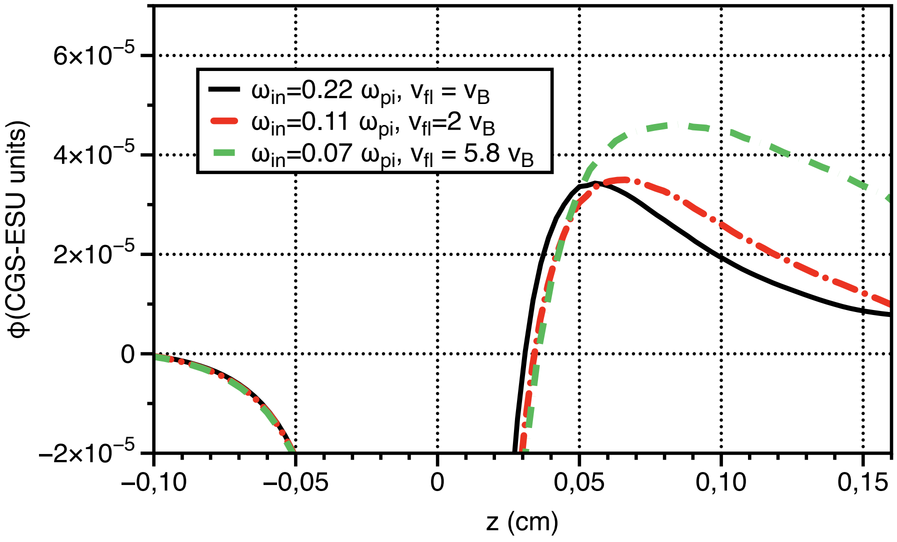

Figure S2. Profiles of the calculated potential distributions around a single dust particle with the charge $Q = 15000e$ along the direction of the external electric field E_0. Three lines correspond to different values of ion-neutral collision frequencies.

In order to describe ion-neutral collisions, we employ the Bhatnagar-Gross-Krook (BGK) operator[22]. This operator assumes that the ion-neutral collision frequency $\omega_{\rm in}$ does not depend on the ion flow rate. $\omega_{\rm in}$ can be calculated from the following formula in CGS-ESU units[23]:

$$\omega_{\rm in} = \frac{P\omega_{\rm pi}\sigma_{\rm in}}{|e|\sqrt{k_B T_n n_i^\infty 4\pi}}, \tag{3}$$

where P is pressure of the parent gas, $\omega_{\rm pi}$ is the ion plasma frequency, T_n is the parent gas temperature, $\sigma_{\rm in} = 10.6\times10^{-19}$ m^2 is the ion-neutral collision cross-section. $\sigma_{\rm in}$ is estimated for Argon gas under the conditions of the experiment according to[16]. The calculated value of ion-neutral collision frequency for the conditions of our experiment is $\omega_{\rm in} = 0.07\omega_{\rm pi}$. The equation (3) only provides a reasonable estimation of the ion-neutral collision frequency in the conditions when BGK collision integral can be employed. At the same time, the variation of this parameter effects significantly the dynamics of dust particles. For this reason, we calculate the wake potentials for three different values of the ion-neutral collision frequency $\omega_{\rm in}$: $0.07\omega_{\rm pi}, 0.11\omega_{\rm pi}, 0.22\omega_{\rm pi}$. The calculated potential profiles for different collision frequencies are shown in the Fig. S2 for the value of grain charge $Q = 15000e$.

Each value of the ion-neutral collision frequency corresponds to a certain value of the ion flow velocity as given by the formula[17]:

$$v_{\rm fl} = \frac{|e|E_0}{m_i\omega_{\rm in}}. \tag{4}$$

For example, at $\omega_{\rm in} = 0.22\omega_{\rm pi}$ the ion flow velocity $v_{\rm fl}$ is equal to the plasma Bohm speed $v_{\rm B}$. With the increase of the ion-neutral collision frequency and the decrease of the ion flow rate, the maximum of the wake potential shifts further downstream from

the parent dust particle. In the main text the wake potential is defined by the values of charge Q and ion flow velocity $v_{\rm fl}$, the values of other parameters are given above.

References

1. Zhdanov, S., Ivlev, A. & Morfill, G. Mode-coupling instability of two-dimensional plasma crystals. *Phys. Plasmas* **16**, 083706 (2009).
2. Melzer, A. Connecting the wakefield instabilities in dusty plasmas. *Phys. Rev. E* **90**, 053103 (2014).
3. Ivlev, A. & Kompaneets, R. Instabilities in bilayer complex plasmas: Wake-induced mode coupling. *Phys. Rev. E* **95**, 053202 (2017).
4. Kryuchkov, N. P. *et al.* Thermoacoustic instability in two-dimensional fluid complex plasmas. *Phys. Rev. Lett.* **121**, 075003 (2018).
5. Zampetaki, A., Huang, H., Du, C.-R., Löwen, H. & Ivlev, A. Buckling of two-dimensional plasma crystals with nonreciprocal interactions. *Phys. Rev. E* **102**, 043204 (2020).
6. Hutchinson, I. H. Ion collection by a sphere in a flowing plasma: I. Quasineutral. *Plasma Phys. Control. Fusion* **44**, 1953 (2002).
7. Hutchinson, I. Collisionless ion drag force on a spherical grain. *Plasma Phys. Control. Fusion* **48**, 185 (2006).
8. Miloch, W. & Block, D. Dust grain charging in a wake of other grains. *Phys. Plasmas* **19**, 123703 (2012).
9. Matthews, L. S. *et al.* Dust charging in dynamic ion wakes. *Phys. Plasmas* **27**, 023703 (2020).
10. Totsuji, H., Kishimoto, T., Totsuji, C. & Tsuruta, K. Competition between two forms of ordering in finite coulomb clusters. *Phys. Rev. Lett.* **88**, 125002 (2002).
11. Schiffer, J. Melting of crystalline confined plasmas. *Phys. Rev. Lett.* **88**, 205003 (2002).
12. Kolotinskii, D. A., Nikolaev, V. S. & Timofeev, A. V. Effect of structural inhomogeneity and nonreciprocal effects in the interaction of macroparticles on the dynamic properties of a dusty plasma monolayer. *JETP Lett.* **113**, 510–517 (2021).
13. Ivlev, A. V., Zhdanov, S., Khrapak, S. & Morfill, G. Ion drag force in dusty plasmas. *Plasma Phys. Control. Fusion* **46**, B267 (2004).
14. Serikov, V. V., Kawamoto, S. & Nanbu, K. Particle-in-cell plus direct simulation monte carlo (pic-dsmc) approach for self-consistent plasma-gas simulations. *IEEE Trans. Plasma Sci.* **27**, 1389–1398 (1999).
15. Grozdanov, T. & Janev, R. Charge-exchange collisions of multiply charged ions with atoms. *Phys. Rev. A* **17**, 880 (1978).
16. Phelps, A. V. Cross sections and swarm coefficients for nitrogen ions and neutrals in n2 and argon ions and neutrals in ar for energies from 0.1 ev to 10 kev. *J. Phys. Chem. Ref. Data* **20**, 557–573 (1991).
17. Kompaneets, R., Morfill, G. E. & Ivlev, A. V. Wakes in complex plasmas: A self-consistent kinetic theory. *Phys. Rev. E* **93**, 063201 (2016).
18. Sundar, S., Kählert, H., Joost, J.-P., Ludwig, P. & Bonitz, M. Impact of collisions on the dust wake potential with maxwellian and non-maxwellian ions. *Phys. Plasmas* **24**, 102130 (2017).
19. Hutchinson, I. Nonlinear collisionless plasma wakes of small particles. *Phys. Plasmas* **18**, 032111 (2011).
20. Allen, J. Probe theory-the orbital motion approach. *Phys. Scr.* **45**, 497 (1992).
21. Gatti, M. & Kortshagen, U. Analytical model of particle charging in plasmas over a wide range of collisionality. *Phys. Rev. E* **78**, 046402 (2008).
22. Else, D., Kompaneets, R. & Vladimirov, S. On the reliability of the bhatnagar–gross–krook collision model in weakly ionized plasmas. *Phys. Plasmas* **16**, 062106 (2009).
23. Ludwig, P., Miloch, W. J., Kählert, H. & Bonitz, M. On the wake structure in streaming complex plasmas. *New J. Phys.* **14**, 053016 (2012).
